# Unsafe care in residential settings for older adults: a content analysis of accreditation reports

**DOI:** 10.1093/intqhc/mzad085

**Published:** 2023-10-26

**Authors:** Peter D Hibbert, Ruby Ash, Charlotte J Molloy, Johanna Westbrook, Ian D Cameron, Andrew Carson-Stevens, Leonard C Gray, Richard L Reed, Alison Kitson, Jeffrey Braithwaite

**Affiliations:** Australian Institute of Health Innovation, Macquarie University, 75 Talavera Rd, North Ryde, Sydney, New South Wales 2109, Australia; IIMPACT in Health, Allied Health and Human Performance, University of South Australia, GPO Box 2471, Adelaide, South Australia 5001, Australia; Anglicare SA, 159 Port Road, Hindmarsh, Adelaide, South Australia 5007, Australia; Australian Institute of Health Innovation, Macquarie University, 75 Talavera Rd, North Ryde, Sydney, New South Wales 2109, Australia; IIMPACT in Health, Allied Health and Human Performance, University of South Australia, GPO Box 2471, Adelaide, South Australia 5001, Australia; Australian Institute of Health Innovation, Macquarie University, 75 Talavera Rd, North Ryde, Sydney, New South Wales 2109, Australia; John Walsh Centre for Rehabilitation Research, Kolling Institute, Northern Sydney Local Health District and Faculty of Medicine and Health, University of Sydney, Reserve Rd, St Leonards, New South Wales 2065, Australia; PRIME Centre Wales & Division of Population Medicine, School of Medicine, Cardiff University, Heath Park, Cardiff, Wales CF14 4YS, UK; Centre for Health Services Research, Faculty of Medicine, The University of Queensland, 288 Herston Road, Herston, Brisbane, Queensland 4006, Australia; Discipline of General Practice, College of Medicine and Public Health, Flinders University, Sturt Rd, Bedford Park, South Australia 5042, Australia; Caring Futures Institute, College of Nursing and Health Sciences, Flinders University, Sturt Rd, Bedford Park, South Australia 5042, Australia; Australian Institute of Health Innovation, Macquarie University, 75 Talavera Rd, North Ryde, Sydney, New South Wales 2109, Australia

**Keywords:** patient safety, homes for the aged, accreditation, classification, quality improvement

## Abstract

Residents of aged care services can experience safety incidents resulting in preventable serious harm. Accreditation is a commonly used strategy to improve the quality of care; however, narrative information within accreditation reports is not generally analysed as a source of safety information to inform learning. In Australia, the Aged Care Quality and Safety Commission (ACQSC), the sector regulator, undertakes over 500 accreditation assessments of residential aged care services against eight national standards every year. From these assessments, the Aged Care Quality and Safety Commission generates detailed Site Audit Reports. In over one-third (37%) of Site Audit Reports, standards relating to Personal and Clinical Care (Standard 3) are not being met. The aim of this study was to identify the types of resident Safety Risks that relate to Personal and Clinical Care Standards not being met during accreditation or re-accreditation. These data could inform priority setting at policy, regulatory, and service levels. An analytical framework was developed based on the World Health Organization’s International Classification for Patient Safety and other fields including Clinical Issue (the issue related to the incident impacting the resident, e.g. wound/skin or pain). Information relating to safety incidents in the Site Audit Reports was extracted, and a content analysis undertaken using the analytical framework. Clinical Issue and the International Classification for Patient Safety–based classification were combined to describe a clinically intuitive category (‘Safety Risks’) to describe ways in which residents could experience unsafe care, e.g. diagnosis/assessment of pain. The resulting data were descriptively analysed. The analysis included 65 Site Audit Reports that were undertaken between September 2020 and March 2021. There were 2267 incidents identified and classified into 274 types of resident Safety Risks. The 12 most frequently occurring Safety Risks account for only 32.3% of all incidents. Relatively frequently occurring Safety Risks were organisation management of infection control; diagnosis/assessment of pain, restraint, resident behaviours, and falls; and multiple stages of wounds/skin management, e.g. diagnosis/assessment, documentation, treatment, and deterioration. The analysis has shown that accreditation reports contain valuable data that may inform prioritization of resident Safety Risks in the Australian residential aged care sector. A large number of low-frequency resident Safety Risks were detected in the accreditation reports. To address these, organizations may use implementation science approaches to facilitate evidence-based strategies to improve the quality of care delivered to residents. Improving the aged care workforces’ clinical skills base may address some of the Safety Risks associated with diagnosis/assessment and wound management.

## Introduction

Residents of aged care services expect safe and effective delivery of quality care. Enquiries and reports across a number of countries[1–4] have highlighted that aged care residents can, and too-often do, suffer safety incidents resulting in harm [‘adverse events’ (AEs)], which can be preventable and serious. Frequently encountered AEs include inadequate wound management and failure to recognize malnutrition and provide nutritional support and over-prescribing [3, 4].

Resident safety incidents in aged care services are less well studied than in acute healthcare [5]. Studies conducted in aged care generally rely on voluntary incident reporting as their data source [6] and often focus on particular incident types such as behaviour [7] or medication [8]. A narrative review on AEs in aged care published in 2022 found that over half the papers focus on only four AE types—deaths, falls, pressure injuries, and fractures [5].

The safety study literature emphasizes that all data sources are subject to reporting biases and tend to capture a particular set of incident types [9]. Capturing multiple information sources is, therefore, important for AEs to be adequately characterized and understood and to facilitate learning and action to reduce further harm to residents. Currently, frequently used sources are incident reports, audits, investigations, and complaints [9].

One of the most frequently undertaken activities to improve the safety and quality of care is accreditation. Accreditation refers to an external peer review that evaluates a healthcare organization’s compliance with predefined performance standards [10]. Narrative information within accreditation reports is not generally analysed as a source of safety information from which to learn. Yet, in principle, such data can have utility at a policy level. In Australia, the Aged Care Quality and Safety Commission (ACQSC), the sector regulator, has undertaken between 311-1222 accreditation assessments per year in the years 2020–2022 from a total of 2705 residential aged care services [11] in the sector to assess the quality of care delivered to residents. The accreditation assessments use the Aged Care Quality Standards (Quality Standards), which are made up of eight overall national standards with 42 substandards or requirements. The accreditation assessors use several information sources including resident, staff, and representative interviews; resident care documentation systems; service documents (e.g. guidelines, forms, charts); risk questions; and observations to assess services. From these assessments, the ACQSC generates confidential and detailed Site Audit Reports, which contain the service’s performance assessment.

The Site Audit Reports assess whether the Quality Standards and requirements have been met [12]. The mostly frequently not met requirements are from Standard 3—Personal and Clinical Care [13] (Supplementary Material, Fig. A1). For site audits undertaken between 1 January 2021 and 31 March 2021, 37% (48 of 129) of facilities did not meet at least one requirement of Standard 3 [13].

The primary goal for aged care services to meet Standard 3 is safety as attested in its Consumer Outcome Statement: ‘I get personal care, clinical care, or both personal care and clinical care, that is safe and right for me’. However, the reasons why services fail these Standard 3 requirements, and the associated Safety Risks to residents outlined in the Site Audit Reports, have not been systematically assessed. This research undertakes an assessment of Site Audit Reports where there was a non-met Standard 3 requirement, using an internationally agreed approach to identifying information within patient safety incidents, the World Health Organization’s (WHO) International Classification for Patient Safety (ICPS). The aim of this study was to identify the types of resident Safety Risks that relate to Personal and Clinical Care Standards not being met during accreditation or re-accreditation. These data in turn could inform safety strategy priority setting and quality improvement at policy, regulatory, organization, and service levels.

## Methods

### Study design and setting

The study undertook a cross-sectional deductive and inductive content analysis of accreditation Site Audit Reports of aged care services in Australia. Aged care services are for senior Australians who can no longer live independently at home and include accommodation and personal care 24 hours a day, as well as access to nursing and general health care services [14]. Services are owned and managed by approved providers [14].

The ACQSC provided the research team with a random sample of 198 Site Audit Reports, assessed between September 2020 and March 2021, of services that had not met at least one Standard 3 requirement. The ACQSC provided Site Audit Reports in the form of Microsoft Word or Adobe Portable Document Format (pdf) using a password-protected secure link.

### Development of an analytical coding framework

The researchers developed an analytical coding framework and database based on the Technical Annex outlined in the WHO ICPS Report [15]. In the WHO ICPS, an incident is defined as an event or circumstance that could have or did lead to harm to a resident [15, 16]. The WHO ICPS class of Incident Types was used to characterize each incident [15, 16], for example, clinical process/procedure, clinical administration, and problems associated with nutrition (Table A1 in the Supplementary Material shows definitions and usage). The ICPS Incident Type class has more granular codes which were used to code the Site Audit Reports, titled ‘Process’ (e.g. screening, diagnosis, and treatment). Table A2 in the Supplementary Material outlines the analytical coding framework. These granular Process codes can classify how care was not delivered optimally, such as problems with assessment or diagnosis, observations not monitored, or escalated when abnormal, and treatment not indicated. No other ICPS domains were used in the analysis due to the nature of the information within the Site Audit Reports.

A field called ‘Clinical Issue’ was added to the analysis of each incident to describe the issue that was impacting the resident (e.g. wound/skin or pain). Clinical Issue codes were inductively developed from the data. The list of Clinical Issues is shown in Table A3 in the Supplementary Material. From a pilot analysis, we ensured that more than one Clinical Issue could be coded per incident in the database.

The information in the Site Audit Reports could be related to an ICPS incident, such as a fall, or more general care, such as managing a resident’s pain (Box 1, Incident 1). If a resident had a fall, the focus was generally not on the fall itself, but on whether the resident was managed appropriately after the fall, or appropriate preventive measures were in place (Box 1, Incident 2). If they were not, then this was recorded as an incident. Similarly, for pain, the focus of the Site Audit Reports was on whether the resident’s pain was managed appropriately (Box 1, Incident 1). Some of the incidents were related to specific residents (Box 1, Incidents 1 and 2), while others were more general hazards with the potential to cause harm (Box 1, Incident 3).

Consistent with previous analyses of safety incidents, more than one incident type can be coded to each incident for chronologically related incidents [17]. Two were pragmatically chosen balancing the acknowledgement that incidents can be complex with the need to analyse and present findings in a practical manner (see Box 1, Incident 4 for an example).

Box 1. **Examples of incidents extracted from Site Audit Reports and codes**

Incident 1: (Resident) has a history of labile (unstable) blood glucose levels (BGLs) and is on regular insulin. Their care plan states to check their BGL post fall to determine if the underlying cause of the fall is sudden change in BGL, which is also congruent with the service’s fall management policy. However, review of BGL charts after last five falls did not indicate that (resident’s) BGL was checked post fall.

**Data source:** Care Document

**Clinical Issue:** Falls management

**Incident type:** Clinical process/procedure

**Process:** Diagnosis/assessment

Incident 2: (Resident) experienced a recent fall resulting in a fracture to their neck of femur. There has been no pain charting completed to assess their pain since (resident) returned to the service on (date).

**Data source:** Care Document

**Clinical Issue:** Pain management

**Incident type:** Clinical process/procedure

**Process:** Diagnosis/assessment

Incident 3: Numerous resident rooms, bathroom and furnishings were observed by the Assessment Team to be covered in grime and dirt and mould in some showers.

**Data source:** Care Document

**Clinical Issue:** Infection control

**Incident type:** Infrastructure/Buildings/Fixtures

**Process:** Buildings/fixtures

Incident 4: (Resident) fed food with the wrong consistency leading to a choking episode. Cardio-Pulmonary Resuscitation was applied to the resident; however, the resident’s status was Not For Resuscitation.

**Data source:** Care Document

**Clinical Issue:** Dysphagia

**Incident type 1:** Nutrition

**Process 1:** Administration

**Incident type 2:** Clinical Process/Procedure

**Process 2:** Procedure/Treatment/Intervention

The sources of data within the Site Audit Reports (i.e. where the information relating to each incident was collected) were identified and coded. These included feedback from residents, their representatives and staff, observations by the assessors, and care and service documents.

### Piloting the data collection form and process

A Microsoft Excel spreadsheet (v2202) was developed to capture data. Test data were initially trialled in the spreadsheet using two Site Audit Reports. During this time, the lead coder (R.A.) and chief investigator (P.D.H.) worked closely to develop specific protocolized rules and examples to apply the classification consistently. Process codes were also added (e.g. clinical deterioration) if they were not represented in the ICPS classification; a second Clinical Issue data field was added that could be used if necessary to code incidents.

### Reviewing and coding the Site Audit Reports

The Site Audit Reports were randomly ordered and then sequentially coded. Coding was undertaken by two experienced aged care nurses. The lead coder (R.A.) was trained in the use of the ICPS and the Clinical Issue field and then trained the subsequent coder. The coders read the sections of the Site Audit Reports that related to Standard 3 not being met. Text describing the incident was extracted and recorded in the database together with the codes.

After review of 65 Site Audit Reports, 2267 incidents were recorded. Data collection was ceased at 65 reports as sufficient data had been collected to characterize the four most frequently occurring incident types based on previous research on incident analysis [18].

### Quality assurance and inter-rater reliability testing

Quality assurance was undertaken during data collection. Weekly meetings between the lead coder and the chief investigator were held during all stages of the project to ensure consistency of the coding process. Monthly meetings were held with a wider project group to report progress and receive feedback. In addition to regular quality assurance, inter-rater reliability assessments were undertaken during data collection.

### Analysis

Clinical Issue and Incident Type/Process were combined to describe a clinically intuitive category (‘resident Safety Risks’ or ‘Safety Risks’), which describes ways in which residents could experience unsafe care. For example, in Box 1, Incident 2, the Safety Risk was ‘Pain management–Clinical process/procedure–Diagnosis/assessment’, which could be shortened to ‘diagnosis/assessment of pain’. Descriptive analysis, presented in frequency distributions, was undertaken. The number of incidents was tabulated separately against Clinical Issue, Incident Type/Process, and the most frequently occurring Safety Risks. Deidentified examples of Safety Risks were also presented.

## Results

The 65 services with Site Audit Reports included in the analysis had a mean of 89.6 (SD 49.7) residential beds; were mainly managed by private (32%), community-based (24%), charitable (21%), or religious (20%) organizations; and were predominantly located in the major cities (60%) or inner regional areas (25%) (Tables A4–A6 in the Supplementary Material). In terms of size, organizational management type, and remoteness, the included services were similar to those services (*n* = 133) not included and across the whole aged care sector (*n* = 2705) [11] (Tables A4–A6 in the Supplementary Material).

**Table 1.** Number of incidents by not met requirement (number of incidents 2267).

Standard 3 requirement	Requirement code	*N* (%)
Safe and Effective Personal and Clinical Care	3.(3)(a)	1,051 (46)
High impact or high prevalence risks managed effectively	3.(3)(b)	596 (26)
End-of-life care	3.(3)(c)	61 (3)
Recognition and response to deterioration	3.(3)(d)	162 (7)
Sharing information to optimize care	3.(3)(e)	150 (7)
Timely and appropriate referrals	3.(3)(f)	36 (2)
Infection risk management and appropriate prescribing	3.(3)(g)	211 (9)
Total		2,267 (100)

Organizations may ‘not meet’ more than one requirement.

From the 65 Site Audit Reports, there were 2267 incidents detected and analysed. There were a mean of 35 incidents per Report (SD: 33) and a median of 25 (interquartile range: 33). The number of incidents per Site Audit Report ranged from 1 to 183 (Fig. A2 and A3 in the Supplementary Material). Kappa scores were assessed within nine Site Audit Reports (comprising 14% of the dataset); there was 0.744 for agreement on the Clinical Issue field, indicating substantial agreement.

The most frequently analysed not met requirements were 3.(3)(a) (Safe and Effective Personal and Clinical Care) and 3.(3)(b) (High impact or high prevalence risks managed effectively) applying to 46% and 26% of incidents, respectively (Table 1). These findings broadly align with the most frequently not met requirements in the summary of Standard 3 across the sector for January—March 2021 (Fig. A1 in the Supplementary Material).

### Data sources for incidents

Seven data sources were identified during review of the Site Audit Reports (Table 2). The most frequently used data source by the assessors was care documents, which contained information on about two-thirds (67%) of the incidents. All other data sources detected fewer than 10% of incidents.

**Table 2.** Data source of incidents (number of incidents 2267).

Data source	Definition	*N* (%)
Care document	Any document that relates directly to care of the resident including care plan, assessments, or clinical directives	1512 (67)
Staff feedback	Feedback provided directly to the assessors by staff during the assessment period	214 (9)
Representative feedback	Feedback provided directly to the assessors by representatives (comprising relatives, friends, or others associated with the resident) during the assessment period	184 (8)
Observation	Any observations that the assessors made on the site during the time of the audit	151 (7)
Service document	Any documents that are classified as procedures, policies, or processes that directly relate to managing the service	148 (7)
Resident feedback	Feedback provided directly to the assessors by residents during the assessment period	58 (3)
Total		2267 (100)

### Clinical Issues, incident types, and Safety Risks

The most frequent Clinical Issues comprising >10% were wound/skin management, infection control, and restraint management (Table 3). The most frequently recorded ICPS incident types/processes were diagnosis/assessment, resources/organisational management, documentation of resident care records, general care/management, and referrals/consultations (Table 4). There were 274 Safety Risks (Clinical Issues and Incident Type/Process combinations) identified. Table 5 outlines the 12 most frequently recorded resident Safety Risks together with deidentified examples. These 12/274 (or 4.4%) Safety Risks account for about one-third (32.3%) of incidents. The most frequently occurring resident Safety Risks were related to management of infection control and diagnosis/assessment of pain, restraint, behaviour, and mobility and falls. Other relatively frequently occurring Safety Risks relate to wounds/skin across multiple stages of the clinical pathway including diagnosis/assessment, documentation, treatment, and deterioration.

**Table 3.** Clinical Issue by number of incidents and frequency and percentage (number of incidents 2267).

Clinical Issue	*N*	%
Wound/skin management	316	13.9
Infection control	248	10.9
Restraint management	236	10.4
Behaviour management	201	8.9
Mobility and falls management	182	8.0
Medication management	179	7.9
Pain management	160	7.1
Health monitoring	95	4.2
Weight management	83	3.7
Medical care	80	3.5
Communication	71	3.1
Care planning	69	3.0
Diabetes management	67	3.0
General care/other	56	2.5
Palliative care	53	2.3
Dietary management	44	1.9
Dysphagia	39	1.7
Safety and risk management	30	1.3
Mental health	28	1.2
Catheter management	19	0.8
Continence care	18	0.8
Consumer needs and preferences	17	0.7
Bowel management	16	0.7
Hygiene care	16	0.7
Staff Behaviour	16	0.7

Total and percentages add to greater than the total number of incidents as each incident could be assigned up to two Clinical Issues.

**Table 4.** Incident type (in bold) and process by frequency and percentage (number of incidents 2267).

Incident type and process	*N*	%
Clinical process/procedure	**1112**	**49.1**
Diagnosis/assessment	487	21.5
General care/management	250	11.0
Procedure/treatment/intervention	132	5.8
Clinical deterioration	121	5.3
Clinical orders	63	2.8
Screening/prevention/routine check-up	27	1.2
Tests/investigations	25	1.1
Specimens/results	4	0.2
Detention/restraint	3	0.1
Documentation	**564**	**24.9**
Charts/medical records/assessments/consultations	258	11.6
Instructions/information/policies/procedures/guidelines	152	6.7
Investigations/incident reports	87	3.8
Forms/certificates	33	1.5
Reports/results/images	13	0.6
Letters/e-mails/records of communication	9	0.4
Checklists	4	0.2
Orders/requests	2	0.1
Resources/organizational management	**324**	**14.3**
Clinical administration	**242**	**10.7**
Referral/consultation	220	9.7
Handover	15	0.7
Appointment	7	0.3
Medication/IV fluids	**97**	**4.3**
Administration	61	2.7
Prescribing	16	0.7
Supply/ordering	9	0.4
Preparation/dispensing	6	0.3
Storage	4	0.2
Delivery	1	0.04
Medical device/equipment	**49**	**2.2**
Nutrition	**28**	**1.2**
Preparation/manufacturing/cooking	8	0.4
Prescribing/requesting	8	0.4
Administration	6	0.3
Dispensing/allocation	3	0.1
Supply/ordering	2	0.1
Delivery	1	0.04
Infrastructure/buildings/fixtures	**23**	**1.0**
Infrastructure/building fixture	21	0.9
Signage	2	0.1
Behaviour	**17**	**0.7**
Healthcare-associated infection (wound)	**3**	**0.1**

Total and percentages add to greater than total number of incidents as each incident could be assigned up to two incident types. As noted in the Method, Process provides more granular details than Incident Type. See Appendix Table A.3 in the Supplementary Material for the modified WHO analytical framework that we used.

**Table 5.** The most frequently occurring 12 resident Safety Risks (a combination of Clinical Issue, Incident Type/Process) (N, %) and resident Safety Risk examples (number of incidents total 2267, n = 732 shown in the table).

Clinical Issue	Incident type and *process*	Examples	*N*	All incidents (%)
Infection control	Resources *Organizational management*	Prepared signage to communicate lockdown/service closure (specific to COVID-19) and to identify areas that are active COVID-19 consumers zone/cohorts are not currently available.	106	4.7
Pain management	Clinical Process/Procedure *Diagnosis/Assessment*	Care notes show that the resident was reviewed (Date), and an Abbey Pain Scale was recommended to be completed by an observer, while staff are attending to their personal care to determine if pain is a reason for residents to react in a certain way. This chart has not been completed.	84	3.7
Restraint management	Clinical Process/Procedure *Diagnosis/Assessment*	The service did not demonstrate regular monitoring of (resident) for signs of distress, harm, and side effects, nor were these provided to the medical practitioner regarding the use of the restraint.	74	3.3
Behaviour management	Clinical Process/Procedure *Diagnosis/Assessment*	While there has been a reduction in the service, psychotropic medication use for the resident’s majority of the resident behaviour relevant to the need for restraint refers to sadness and isolation. However, a review of their behaviour care plan does not support individualized goals and strategies have been considered and applied.	69	3.0
Mobility and falls management	Clinical Process/Procedure *Diagnosis/Assessment*	At (time), (resident) was found in the lounge area having experienced an unwitnessed fall, sustaining injury to their face with bleeding nose and mouth, and voicing complaint of left hand and neck pain. Blood pressure, respiration, and pulse and oxygen saturation observations were recorded in progress notes to be within normal ranges; however, no documentation on physical and neurological observation charts was completed.	67	3.0
Wound and skin management	Clinical Process/Procedure *Diagnosis/Assessment*	Staff noted (resident’s) foot to be red and swollen with ‘red streaks running up her leg’. Care staff removed the dressing and found resident’s toe to be inflamed and ‘looked infected’. The Medical Officer was notified; however, a documented wound assessment or incident report was not completed at this time. and no treatment plan commenced.	60	2.6
Medication management	Medication/Intravenous fluids *Administration*	The Assessment Team noted on the medication chart (resident) was administered 1 mg on six occasions between (Date 0) and (Date 1), which is 0.5 mg more than the prescribed dose.	53	2.3
Wound and skin management	Documentation *Care records*	Multiple photos of the wounds recorded in the resident’s charts were of poor quality and do not show the full representation of how the wound has deteriorated.	50	2.2
Wound and skin management	Clinical Process/Procedure *Procedure/Treatment/Intervention*	(Resident’s) wound is to be attended to daily. The Assessment Team noted that during the month of (month), the wound was not attended on the following dates: 1, 2, 6, 8, 12, 13, 15, 16, 18, 19, 21, 22, 24, 25, 28, and 29.	48	2.1
Wound and skin management	Clinical Process/Procedure *General Care/management*	The Assessment Team observed that (resident) was resting in a comfort chair, which was not appropriately sized for his height. Observations included (residents) lower legs hanging beyond the support area of the equipment.	41	1.8
Restraint management	Documentation *Care Records*	The Assessment Team identified that no behavioural charting or assessment process is conducted as part of the psychotropic review.	40	1.8
Wound management	Clinical Process/Procedure *Deterioration*	Wound photographs show that the wound continued to deteriorate, and by (date), there was a large necrotic ulcer. The most recent photograph of this wound dated (date) shows that the ulcer remains necrotic with some sloughy areas and is possibly 4 cm in diameter.	40	1.8

## Discussion

### Statement of principal findings

Of the 2267 incidents detected across 65 Site Audit Reports, the most frequent Clinical Issues were the management of wound/skin, resident behaviours and restraint, and infection control. The 12 or 4.4% most frequent Safety Risks accounted for approximately one-third of all incidents. The six most frequently occurring resident Safety Risks were management of infection control and diagnosis/assessment of pain, restraint, behaviour, mobility and falls, and wound/skin. Infection control issues were relatively frequent, being involved in about 11% of incidents. This was likely to be due to increased requirements to comply with process changes due to the coronavirus disease (COVID-19) pandemic.

### Strengths and limitations

There are no previous studies using accreditation reports to characterize safety in the literature to our knowledge. The strength of the study was in using the combination of an established conceptual framework for safety, the ICPS, as well as an inductive framework (related to Clinical Issues) specific to the research objectives. Using a low number of nurses (two) to classify the Site Audit Reports potentially reduces variation in interpretation of the Site Audit Reports. Rigorous continuous quality assurance approaches to the coding were applied, and inter-rater reliability measured was substantial.

The Site Audit Reports were designed to enable the ACQSC to decide whether providers have complied with the Aged Care Quality Standards. They are not designed as sources of resident safety incidents, and some of the descriptions were brief; thereby, it may not be possible to fully understand the context in which they occurred. The interpretation of accreditation assessors in relation to the criteria for meeting Aged Care Quality Standards may differ and impact the underlying Site Audit Reports that were the data source for this study.

### Interpretation within the context of the wider literature

The findings from our study show a much more diverse set of risks that affect the safety of residents than many previous studies, which often use incident reporting as the data source [5]. The risks that our study identified include, for example, infection control and management of restraint, behaviour, medication, pain, and weight. This underscores the importance of capturing multiple information sources to adequately characterize Safety Risks [9].

We could find no studies that systematically analysed safety incidents in relation to Incident Types and Process in aged care. While problems with resources and documentation of resident care records are well known in most care settings including aged care services [4], a unique contribution of this study is finding significant problems with the clinical process of diagnosis/assessment most frequently related to the management of pain, restraint, behaviour, mobility and falls, and wounds/skin, which collectively comprise one in six Safety Risks (15.6%). Our findings of the most frequent clinical administration problems to be timing and appropriate referrals/consultations to specialist clinicians and services are also not well recognized in the literature.

### Implications for policy, practice, and research

One consistent frustration of the safety sector is that much effort is expended on collecting data; however, there is not enough time dedicated to analysis and sense-making [19]. The accreditation regulator and data custodian, the ACQSC, should be commended for recognizing the potential value of the Site Audit Reports and making them securely available for analysis. We encourage similar organizations holding data that can potentially inform safety to a similar commitment to learning.

The analysis shows that at the policy or systems level, the Site Audit Reports contain valuable data with an average of 35 incidents detected per Report. The information may assist in prioritization of the main Clinical Issues and types of safety problems that are occurring in the Australian residential aged care sector. If a similar analysis was to be conducted on underlying accreditation reports in other countries or services (e.g. healthcare), an assessment would need to be made of the structure and content of them to ensure that they are indeed informative in relation to safety. This means that they not only are likely to be qualitative, detailed (the relevant sections of the ACQSC Site Audit Reports were on average 15 pages, but can be up to 50 pages), and contain information from the resident care record (which was the source of two-thirds of the incidents in this study—Table 2) but also contained multiple information sources (6/7 sources provided one-third of the incidents).

One of the main findings of this analysis is that there are a large number of infrequently occurring resident Safety Risks. Considering the data at the level of Clinical Issue (Table 3) shows a similar profile of many infrequently occurring issues, with the least frequently occurring 22 of the 25 Clinical Issues making up over two-thirds (67.7%) of incidents. A similar distribution of safety incidents has been found in healthcare [20]. The large number of low-frequency issues illustrates the complex nature of caring for aged care residents and provides a challenge to services and organizations to achieve higher quality of care for their residents as targeting each individual issue requires significant resources and opportunity costs. Even sourcing credible and accessible evidence for what works for each of these Clinical Issues can be challenging, particularly for smaller organizations.

Instead of solely focusing on improving the myriad of individual Safety Risks, aged care services and organizations may consider implementing evidence-based overarching strategies, which can improve the overall safety and quality of care delivered to residents, thereby addressing many Clinical Issues in parallel. Examples of such strategies include safety culture and leadership, co-design with residents, high reliability teams, structured handover and communication, electronic clinical systems, clinical design support, and locally agreed protocols based on evidence (clinical pathways) [21–28]. The evidence for these strategies is mainly in healthcare, and their applicability to aged care needs further work. Their strategic adoption in aged care, underpinned by organizational-level sustainable quality improvement systems [29] and implementation science [30], is likely to facilitate implementation of high-evidence strategies for improving the safety and quality of care to residents. Ultimately, an end goal is a learning aged care system—one where science, informatics, incentives, and culture are aligned for enduring continuous improvement and innovation [31–33] and where effective governance supports learning.

The number and complexity of resident Safety Risks identified in this analysis, and in particular, those relating to diagnosis/assessment, referral/consultations, and wound management, suggest that improving the clinical skills base of the aged care workforce may be warranted. A policy recommended by a recent Australian Royal Commission into Quality and Safety in Aged Care [4] mandating the presence of a registered nurse 24 h per day in aged care services is currently being implemented, which may address some of the issues identified. Further analyses 12 months after this policy change might provide evidence of its effect.

## Conclusion

At the policy or systems level, narrative information within accreditation reports may contain valuable data to prioritize the main Clinical Issues and types of safety problems occurring in the Australian aged care sector. A wide array of relatively low-frequency Clinical Issues was detected within the narrative information in aged care accreditation reports. To effectively tackle this wide array of Clinical Issues, organizations may consider implementing evidence-based overarching strategies, which can improve the overall safety and quality of care delivered to residents, thereby addressing many Clinical Issues in parallel.

## Supplementary Material

mzad085_Supp

## Data Availability

Due to the sensitive nature of the data and the need to manually deidentify text, data are not available for further analysis.
