# Supplementary material for: Unsafe care in residential settings for older adults: a content analysis of accreditation reports
Source: Int J Qual Health Care. 2023 Oct 26;35(4):mzad085. doi: 10.1093/intqhc/mzad085 (PMC10654691; doi:10.1093/intqhc/mzad085)
Supplement: mzad085_Supp [file mzad085_supp.zip › suppl_data/Supplementary data.docx]

**Supplementary material**

*Figure A1: Standard 3 – Personal and Clinical Care non-met requirements January-March 2021*

*Modified from reference (9) in the main paper: Aged Care Quality and Safety Commission. Residential Care Sector Performance January-March 2021. 2021. Available from:* [*www.agedcarequality.gov.au/sites/default/files/media/acqsc-sector-performance-data-january-march-2021.pdf*](https://mymailunisaedu-my.sharepoint.com/personal/hibberpd_unisa_edu_au/Documents/Documents/MU/Papers/CT%20AGED/www.agedcarequality.gov.au/sites/default/files/media/acqsc-sector-performance-data-january-march-2021.pdf)

Table A1: ICPS incident types, definitions and how they were used

| **Incident type** | **Definitions and usage** |
| --- | --- |
| Behaviour (Beh/staff/pt) | Behaviour incidents incorporated both staff and resident behaviours. Of the incidents identified in the audits most involved staff, including staff using a lifter incorrectly resulting in a resident injury, staff lying about the cause of the resident’s injuries, staff being rude to residents and staff betting on the time of death for a resident. |
| Blood / blood products (Bl) | There were no incident types in this category in the reports audited |
| Clinical administration (CA) | Clinical administration incidents include failures to ensure that a referral was done, followed up on or actioned in a timely manner. |
| Clinical process/procedure (CPP) | Clinical process / procedure incidents are associated with resident clinical care problems comes from the non-delivery, incomplete or failure to follow established processes and procedures. |
| Documentation (DocInv) | Documentation incidents involve a failure in one of the following: the absence of an assessment, no review of an assessment when residents’ needs changed, information missing from documents or staff have not followed the care plan. |
| Falls (Falls) | There were no incident types in this category in the reports audited |
| Healthcare associated infections (HAISite) | Are specific site and pathogen infections (noting that there were incidents for this incident type which were all maggot infested wounds). |
| Infrastructure/ building / fixtures (Infra) | Infrastructure / building / fixtures indicates issues with condition and cleanliness of areas including resident’s rooms. |
| Medical / device / equipment (Device) | Equipment was found to be lacking in safety, resident suitability, and availability |
| Medication / IV fluids (Med) | Medications / IV fluid indicates there was a failure to administer the correct medication dose, the correct medication, medications out of date, medications given without an order, medications given by unqualified staff member, missing information from medication chart and incorrect medication management by staff. |
| Nutrition (Nut) | Nutrition indicates that residents were not delivered the prescribed diet including consistency of diet, staff were not aware of residents specialised dietary needs, dietary details incorrect, residents received food they did not like, and residents not assisted with meals. |
| Oxygen / gas / vapour (O2) | There were no incident types in this category in the reports audited |
| Patient accidents (Acc) | There were no incident types in this category in the reports audited |
| Resource / organisation / management (Reso) | Resources / organisational management involve a failure to have in place service documents, supply the correct level of staffing or skill mix or that staff are suitably educated. |

Table A2: Analytical Coding Framework – modified International Classification for Patient Safety (ICPS). I

| **Incident Type** | **Process** |
| --- | --- |
| Clinical Administration | Handover |
|  | Appointment |
|  | Waiting list |
|  | Referral/Consultation |
|  | Admission |
|  | Discharge |
|  | Transfer of care |
|  | Patient identification |
|  | Consent |
| Clinical Process and Procedure | Screening/prevention/ routine check-up |
|  | Diagnosis/assessment |
|  | Procedure/ treatment/ intervention |
|  | General care/management |
|  | Tests/investigations |
|  | Specimens/results |
|  | Detention/restraint |
|  | Clinical orders |
|  | Deterioration |
| Documentation | Orders/requests |
|  | Charts/medical records/ assessments/ consultations |
|  | Check lists |
|  | Forms/certificates |
|  | Instructions/information/policies/procedures/guidelines |
|  | Labels/Stickers/Identification Bands/Cards |
|  | Reports/results/images |
| Healthcare Associated Infection | Bloodstream |
|  | Surgical site |
|  | Abscess |
|  | Respiratory |
|  | Intravascular cannulae |
|  | Infected prosthesis/site |
|  | Urinary drain/tube |
|  | UTI |
|  | Cellulitis |
|  | Conjunctivitis |
|  | VRE |
|  | MRSA |
|  | Gastroenteritis |
|  | Wound |
| Medication/IV Fluids | Prescribing |
|  | Preparation/dispensing |
|  | Presentation/packaging |
|  | Delivery |
|  | Administration |
|  | Supply/ordering |
|  | Storage |
|  | Monitoring |
| Blood/Blood Products | Pre-Transfusion Testing |
|  | Prescribing |
|  | Preparation/Dispensing |
|  | Delivery |
|  | Administration |
|  | Storage |
|  | Monitoring |
|  | Presentation/Packaging |
|  | Supply/Ordering |
| Nutrition | Prescribing/Requesting |
|  | Preparation/Manufacturing/ Cooking |
|  | Supply/Ordering |
|  | Presentation |
|  | Dispensing/Allocation |
|  | Delivery |
|  | Administration |
|  | Storage |
| Oxygen/Gas/ Vapour | Cylinder Labelling/Color Coding/PIN Indexing |
|  | Prescription |
|  | Administration |
|  | Delivery |
|  | Supply/Ordering |
|  | Storage |
| Medical device/ equipment | Medical device/equipment |
| Behaviour | Staff/Pt Behaviour |
| Patient accident | Pt accident |
| Falls | Fall involving cot |
|  | Fall involving bed |
|  | Fall involving chair |
|  | Fall involving stretcher |
|  | Fall involving toilet |
|  | Fall involving therapeutic equipment |
|  | Fall involving stairs/steps |
|  | Fall involving being Carried/Supported by Another Individual |
| Infrastructure/Buildings/Fixtures | Infrastructure/building fixture |
|  | signage |
| Resources/Organisational management | Resources/organisational management |

Table A3: List of Clinical Issues inductively developed from the data

| **Clinical Issue** |
| --- |
| Behaviour Management |
| Bowel management |
| Care planning |
| Catheter management |
| Communication |
| Consumer needs and preferences |
| Continence care |
| Diabetes management |
| Dietary Management |
| Dysphagia |
| General care / other |
| Health monitoring |
| Hygiene Care |
| Infection control |
| Medical care |
| Medication management |
| Mental health |
| Mobility and Falls management |
| Pain management |
| Palliative care |
| Restraint management |
| Safety and Risk management |
| Staff Behaviour |
| Weight management |
| Wound / skin management |

*Table A4: Number of Residential Places of all residential aged services in Australia, services with Site Audit Reports included in the analysis, and services with Site Audit Reports not included in the analysis*

|  | Site Audit Reports included in the analysis (n=65) | Site Audit Reports not included in the analysis (n=133) | All residential aged services in Australia (n=2,705) |
| --- | --- | --- | --- |
| Average | 89.6 (49.7) | 82.9 (SD 41.1) | 81 (SD 43.3) |
| Range | 15 - 293 | 10 - 173 | 2 - 333 |

SD: Standard deviation

*Table A5: All residential aged services in Australia, services with Site Audit Reports included in the analysis, and services with Site Audit Reports not included in the analysis by Organisation Type (n, %)*

| Organisation Type | Site Audit Reports included in the analysis (n, %) | Site Audit Reports not included in the analysis (n, %) | All residential aged services in Australia (n, %) |
| --- | --- | --- | --- |
| Private Incorporated Body | 21 (32) | 51 (38) | 931 (34) |
| Religious | 13 (20) | 25 (19) | 611 (23) |
| Charitable | 14 (21) | 25 (19) | 515 (19) |
| Community Based | 16 (24) | 27 (20) | 412 (15) |
| State Government | 1 (2) | 3 (2) | 208 (8) |
| Local Government | 0 (0) | 2 (2) | 25 (1) |
| Other | 0 (0) | 0 (0) | 3 (<1) |
| **TOTAL** | **65** | **133** | **2705** |

*Table A6: All residential aged services in Australia, services with Site Audit Reports included in the analysis, and services with Site Audit Reports not included in the analysis by Australian Bureau of Statistics Remoteness Index (n, %)*

| Australian Bureau of Statistics Remoteness Index | Site Audit Reports included in the analysis (n, %) | Site Audit Reports not included in the analysis (n, %) | All Residential Aged services in Australia (n, %) |
| --- | --- | --- | --- |
| Major Cities of Australia | 39 (60) | 74 (56) | 1695 (63) |
| Inner Regional Australia | 16 (25) | 32 (24) | 650 (24) |
| Outer Regional Australia | 10 (15) | 25 (19) | 318 (12) |
| Remote Australia | 0 (0) | 2 (2) | 32 (1) |
| Very Remote Australia | 0 (0) | 0 (0) | 10 (<1) |
| TOTAL | 65 | 133 | 2705 |

Figure A2: Frequency distribution – number of incidents per service (Site Audit Report)


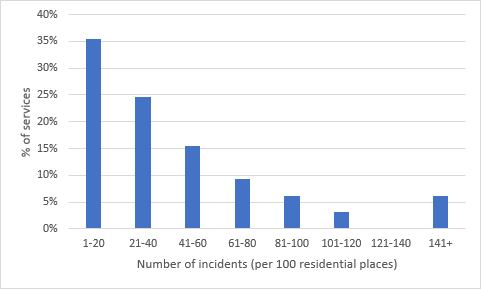


Figure A3: Frequency distribution – number of incidents per 100 residential aged care beds by service (Site Audit Report) (%)
